# A Broad G Protein-Coupled Receptor Internalization Assay that Combines SNAP-Tag Labeling, Diffusion-Enhanced Resonance Energy Transfer, and a Highly Emissive Terbium Cryptate

**DOI:** 10.3389/fendo.2015.00167

**Published:** 2015-11-09

**Authors:** Angélique Levoye, Jurriaan M. Zwier, Agnieszka Jaracz-Ros, Laurence Klipfel, Martin Cottet, Damien Maurel, Sara Bdioui, Karl Balabanian, Laurent Prézeau, Eric Trinquet, Thierry Durroux, Françoise Bachelerie

**Affiliations:** ^1^INSERM U1148, Laboratory of Vascular Translational Science, Université Paris 13, Sorbonne Paris Cité, Paris, France; ^2^Cisbio Bioassays, BP84175, Codolet, France; ^3^INSERM UMR996, Inflammation, Chemokines and Immunopathology, Université Paris-Sud, Université Paris-Saclay, Clamart, France; ^4^CNRS UMR 5203, INSERM U1191, Institut de Génomique Fonctionnelle, Université Montpellier 1 & 2, Montpellier, France

**Keywords:** GPCRs, Internalization, DERET, HTRF, CXCR4, CXCR7, V_1a_, Lumi4-Tb

## Abstract

Although G protein-coupled receptor (GPCR) internalization has long been considered as a major aspect of the desensitization process that tunes ligand responsiveness, internalization is also involved in receptor resensitization and signaling, as well as the ligand scavenging function of some atypical receptors. Internalization thus contributes to the diversity of GPCR-dependent signaling, and its dynamics and quantification in living cells has generated considerable interest. We developed a robust and sensitive assay to follow and quantify ligand-induced and constitutive-induced GPCR internalization but also receptor recycling in living cells. This assay is based on diffusion-enhanced resonance energy transfer (DERET) between cell surface GPCRs labeled with a luminescent terbium cryptate donor and a fluorescein acceptor present in the culture medium. GPCR internalization results in a quantifiable reduction of energy transfer. This method yields a high signal-to-noise ratio due to time-resolved measurements. For various GPCRs belonging to different classes, we demonstrated that constitutive and ligand-induced internalization could be monitored as a function of time and ligand concentration, thus allowing accurate quantitative determination of kinetics of receptor internalization but also half-maximal effective or inhibitory concentrations of compounds. In addition to its selectivity and sensitivity, we provided evidence that DERET-based internalization assay is particularly suitable for characterizing biased ligands. Furthermore, the determination of a *Z*′-factor value of 0.45 indicates the quality and suitability of DERET-based internalization assay for high-throughput screening (HTS) of compounds that may modulate GPCRs internalization.

## Introduction

G protein-coupled receptors (GPCRs) are membrane proteins that respond to a wide variety of extracellular stimuli and play critical roles in intercellular communication (1). They are central to many physiological and pathological processes and represent one of the most important classes of drug targets. Classically, agonist-induced GPCR activation results in the activation of heterotrimeric G proteins and the accumulation of second messengers such as Ca^2+^, cyclic AMP, or inositol phosphates. During continuous agonist stimulation, GPCR activation diminishes through a process known as desensitization, which operates directly at the receptor and downstream. Desensitization is typically controlled by receptor phosphorylation mediated by kinases, including the family of GPCR kinases, which promotes the recruitment of β-arrestins to the receptor. In many cases, these processes also lead to receptor internalization, which generally proceeds by either a clathrin-coated pit or caveolae-mediated pathway (2). However, GPCR internalization is also known to promote receptor-mediated signaling and resensitization. Indeed, recent studies have shown that internalized GPCRs can continue to either stimulate or inhibit cAMP production in a sustained manner (3). Moreover, internalization also participates in the ligand-scavenging function of atypical GPCRs that constitutively cycle between the plasma membrane and intracellular compartments (4). Receptor internalization is a ubiquitous process and an indicator of activation for a wide variety of GPCRs including orphan receptors. Thus, internalization contributes to the diversity of GPCR-dependent signaling pathways and its dynamics in living cells has generated considerable interest. Although fluorescence microscopy permits the disappearance of labeled proteins from the cell membrane to be visualized, signals from labeled internalized receptors in intracellular compartments can also be detected, which can bias observations (5). Other techniques for directly measuring receptor internalization include Enzyme-Linked immunoabSorbent Assay (ELISA) and flow cytometry (6), but they are poorly suited for dynamic analyses due to the numerous washing steps involved in these methods, notably harsh treatments (e.g., acidic wash) to dissociate ligands from their receptors. Moreover, because only a small fraction of the receptors at the cell surface for any particular type GPCR undergo internalization, methods must be selective and sensitive to be precise. Hence, sensitive plate-based assays with high signal-to-background ratios have appeared for quantifying β-arrestin recruitment using bioluminescence resonance energy transfer (BRET) technology (7) or enzyme fragment complementation (8, 9) or for determining the extent of receptor internalization by monitoring changes in fluorescence resonance energy transfer (FRET) (10). Additionally, alternative strategies based on the quenching of internalized receptor tagged with a pH-sensitive fluorescent protein probes (11), NanoLuc^®^ luciferase (12), or fluorogen activating protein (FAP) have also been used to monitor internalization (13).

Here, we extended the use of a single labeling method, which displays the requested sensitivity compatible with high-throughput screening (HTS) for the dynamic and quantitative analysis of GPCR internalization. This diffusion-enhanced resonance energy transfer (DERET) method relies on the use of a luminescent terbium cryptate derivative that irreversibly labels cells expressing SNAP-tagged (ST) GPCRs. The ST is a derivative of O^6^-guanine nucleotide alkyltransferase, which can covalently react with fluorescent-conjugated benzyl guanine substrates (14). To validate the DERET as a reliable and quantitative assay for analysis of constitutive- and ligand-induced receptor internalization, we compared it to classic techniques for various GPCRs from the class A such as the vasopressin V_1a_ and δ opioïd receptors and the two receptors of the CXCL12 chemokine (CXCR4 and ACKR3/CXCR7; named CXCR7 throughout the text), and from the class C such as the metabotropic glutamate receptors 5 (mGluR5) receptors.

## Materials and Methods

### Reagents, Plasmids, and Cell Lines

The Tag-lite^®^ labeling medium (catalog reference LABMED) and SNAP-Lumi4^®^-Tb (catalog reference SSNPTBX) SNAP-tag (ST) fusion labeling system were conceived and synthesized by Cisbio Bioassays (Codolet, France). The 96-well plates and 384 small volume-well plates were purchased from Greiner Bio-One (Monroe, NC, USA). CXCL12 was provided by Dr. F. Baleux (Institut Pasteur, Paris, France). Arginine-Vasopressin (AVP) was from Bachem and SR49059 was a gift from Sanofi. Naloxone, SNC162, and SNC80 were obtained from Tocris Bioscience (Bristol, UK). Naltrindole, naltrexazone, methadone, morphine, DAMGO, glutamate, fluorescein, AMD3100 and poly-l-ornithine (MW of 30,000–70,000 Da) were from Sigma-Aldrich (St. Louis, MO, USA). Chalcone 4 was a gift from Dr. Bonnet, UMR7200, University of Strasbourg, Illkirch, France. Lipofectamine 2000 was purchased from Life Technologies (Cergy Pontoise, France). To obtain pRK5–Flag-ST–CXCR7 and –CXCR4 plasmids, the respective coding sequences in the pTRIP–CXCR7 and pTRIP–CXCR4 vectors (15) were amplified by PCR and inserted in the pRK5–Flag-ST–GB2 plasmid (gift from Dr. Pin, Institut de Génomique fonctionnelle, Montpellier, France) previously digested with *Mlu*I and *Hin*dIII to remove the GB2 coding sequence. The plasmid encoding the Flag-ST human CXCR7ΔCter truncated of 41 C-terminal residues (Δ322–363) was generated and provided by Cisbio Bioassays. HEK-293 cells stably expressing ST CXCR4 (catalog reference C1SU1CXCR4) and ST CXCR7 (catalog reference C1SU1CXCR7), ST V_1a_ (catalog reference C1PU1V1A), ST V_2_ (catalog reference C1PU1V2) and CHO cells stably expressing ST δ opioïd receptor (catalog reference C2SU1DOP) were provided by Cisbio Bioassays. ST-receptors constructs used in this study have been described in previous reports and showed no alteration of their pharmacological and functional properties (14, 16–18).

### Cell Culture and Transient Transfections

For the analyses of CXCR4, CXCR7, V_1a_, mGluR5, and δ opioid receptors, HEK-293 or CHO cells were grown at 37°C, 5% CO_2_ in complete culture medium (Dulbecco’s Modified Eagle’s Medium or HAM-F12 Medium, respectively, supplemented with 10% (*v*/*v*) fetal bovine serum, 4.5 g/l glucose, 100 U/ml penicillin, 0.1 mg/ml streptomycin, 1 mM glutamine, and 20 mM HEPES) (all reagents were obtained from Life Technologies). Cells stably expressing ST receptors were grown in complete culture medium supplemented with 0.6 mg/ml geneticin. Transient expression in HEK-293T cells was achieved using the transfection reagent, FuGene 6 (Roche, Basel, Switzerland) or by reverse transfection using Lipofectamine 2000 (Life Technologies). Briefly, for the reverse transfection, 96-well plates were coated with poly-l-ornithine (50 μl of 10 mg/ml) for 30 min at 37°C. After washing, transfection mixes were added to the plate and pre-incubated for 20 min at room temperature. Then, 1–2 × 10^3^ cells were plated in each well and incubated at 37°C under 5% CO_2_ for 24 h.

### DERET Internalization Assay

Internalization assays for CXCR4 and CXCR7 were performed in 96-well culture cell plates using HEK-293T cells as described above. For transient transfection, cell culture medium was removed 24 h after transfection and 100 nM of SNAP-Lumi4-Tb previously diluted in Tag-lite labeling medium was added (50 μl per well) and further incubated for 1 h at 4°C. Excess SNAP-Lumi4-Tb was removed by washing each well four times with 100 μl of Tag-lite labeling medium. Internalization experiments were performed by incubating cells with Tag-lite labeling medium, either alone or containing CXCL12, in the presence of fluorescein. Typically, in plates containing SNAP-Lumi4-Tb-labeled cells, 50 μl of medium containing CXCL12 at the indicated concentrations was added, immediately followed by the addition of 50 μl of 48 μM fluorescein. ST-V_1a_ receptor internalization assay was monitored as described below. ST-δ opioïd receptor internalization was monitored at room temperature on a CHO cell line stably expressing the receptor in either 96-well plates (1 × 10^5^ adherent cells per well) or 384 small volume well plates (5 × 10^3^ cells in suspension per well). ST-δ opioid receptor labeling and internalization measurement following addition of δ opioïd ligands (the agonists SNC162, SNC80, morphine, methadone, and the antagonists naloxone, naltrindole, and naltrexazone) but also μ opioïd ligand such as the agonist DAMGO (17) were done as described above.

### V_1a_ Labeling, Expression, Internalization, and Recycling at the Cell Surface

Surface expressed V1a in HEK-293 stably expressing the receptor were labeled with SNAP-Lumi4-Tb substrate for 1 h at 4°C. The excess of SNAP-Lumi4-Tb was then removed by washing each well three times with Tag-lite labeling medium. For kinetic internalization experiments, cells were stimulated with 1 μM and receptor expression levels determined at 37°C by measuring the SNAP-Lumi4-Tb fluorescence intensity at 620 nm over time. Dose–response experiments were performed on cells in the presence of increasing concentrations of AVP. Receptor expression levels were then evaluated after 1 h of incubation at 37°C. Real-time recycling of V_1a_ was analyzed by stimulating cells with the agonist AVP and the antagonist SR49059. Forty-five minutes following AVP addition, SR49059 was added to block receptors internalization and to detect any recycled ST-V_1a_ receptors at the cell surface.

### Fluorescence Microscopy

Cells expressing ST-δ receptor and ST–CXCR7 expressing cells were seeded in 8-well slides for 24 h. Cell surface ST-δ receptor molecules were labeled during for 30 min with the cell-impermeable O^6^-benzylguanine derivative cell impermeable SNAP-Alexa Fluor 488 (New England Biolabs, Ipswich, MA, USA) at room temperature. Cells were washed three times with Krebs/Tris buffer and incubated for 30 min with either Tag-lite medium either alone or containing SNC162 (10 μM) or naloxone (10 μM). ST–CXCR7 cells were labeled for 1 h with SNAP-red (Cisbio Bioassays) at 4°C and then warmed to 37°C for 1 h to visualize constitutive internalization. Fluorescence was detected on a Zeiss fluorescent microscope with a xenon flash lamp using a GFP or Cy5 filter cube and a Zeiss Plan Apo 63× (1.40 oil) objective.

### Data Presentation and Analysis

Signals emitted at 620 and 520 nm were collected at the indicated times using time-resolved settings for the donor (1500 μs delay, 1500 μs reading time) and acceptor (150 μs delay, 400 μs reading time). Ratio 620/520 (*R*) was obtained by dividing the donor signal (620 nm) by the acceptor signal (520 nm) at a chosen time and multiplying this value by 10,000. Data are expressed as percent of maximal internalization [“% of max. internalization (620/520 nm)”] and were calculated using the following formula: [(*R*_t_ – *R*_min_)/(*R*_max_ – *R*_min_)] × 100 where *R*_t_ corresponds to the ratio observed at a chosen time. Dose–response curves were fitted using non-linear regression dose–response “log [ligand] versus responses (with three parameters except for CXCR7)” routine of Prism 5 (GraphPad Software, La Jolla, CA, USA) to provide the maximal (*E*_max_) and half maximal effective concentration EC_50_ values. The half-time (*t*_1/2_) determination for internalization of the V_1a_ receptor was made by fitting data using a single-phase exponential decay equation with Prism 5 (GraphPad Software, La Jolla, CA, USA).

## Results and Discussion

**Figure 1:**
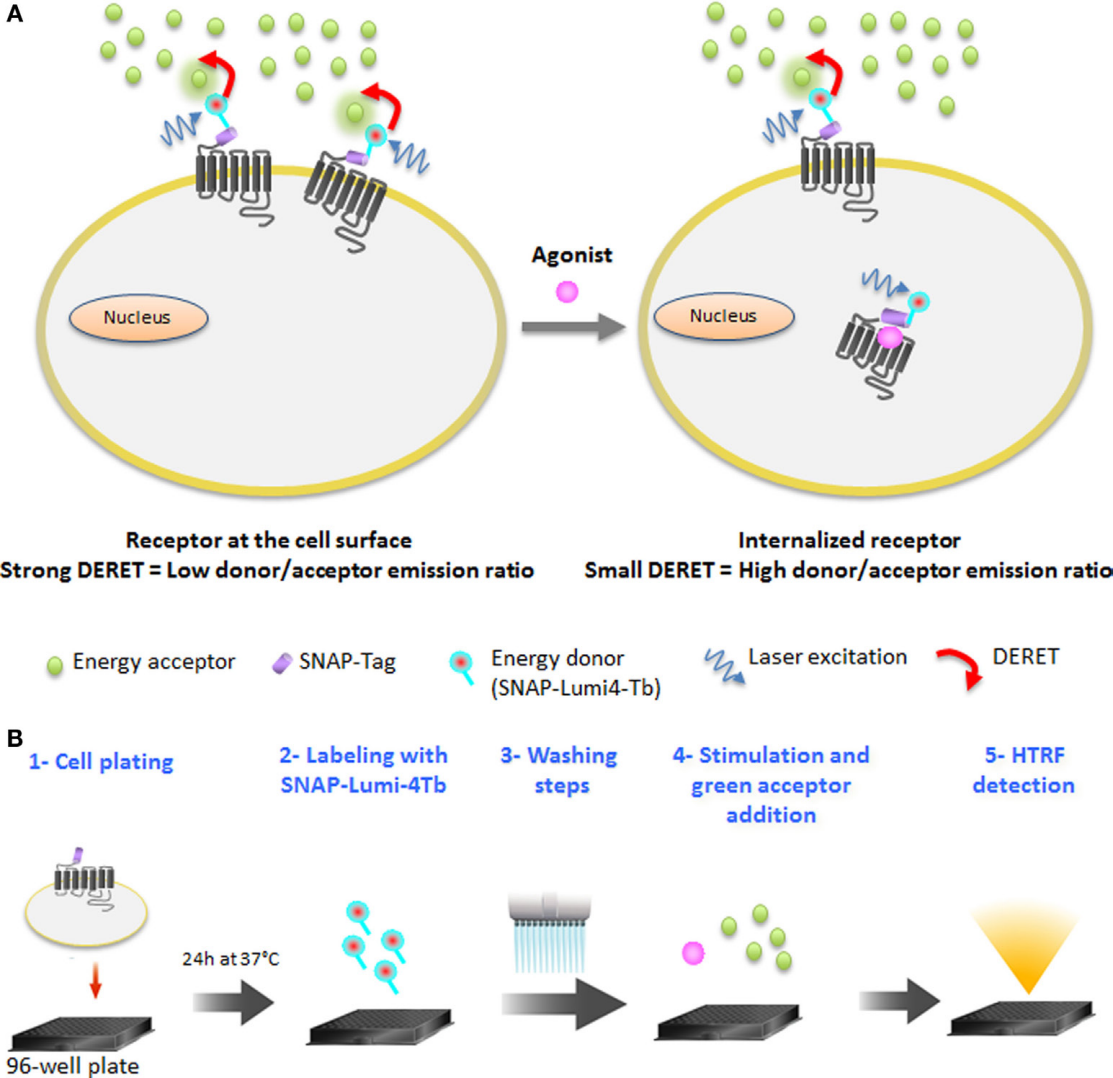
**DERET Internalization assay principle and protocol**. **(A)** GPCRs at the cell surface and bearing the SNAP-Tag are labeled covalently with cell-impermeable SNAP-Lumi4^®^-Tb (energy donor, blue). When the receptor is at the cell surface, addition of a free energy acceptor (green dots) in micromolar concentrations to the cell medium leads to efficient energy transfer, quenching the donor luminescence and resulting in a high DERET signal (left). Following constitutive or agonist-induced internalization of the receptor, energy transfer to the acceptor is reduced, increasing the lifetime of the donor and hence decreasing the DERET (right). **(B)** The protocol of DERET-based internalization assay includes five steps: cell plating (step 1), cell membrane receptors labeling with SNAP-Lumi4-Tb (step 2), washing steps (step 3) to remove the substrate in excess, agonist stimulation, and fluorescein acceptor addition (step 4), and HTRF measurement (step 5) as described in the Section “Materials and Methods.”

In this study, we used a time-resolved FRET-based receptor internalization assay to characterize the dynamics of vasopressin, opioïd, chemokine, and glutamate receptors internalization in real-time in response to different ligand concentrations. Lanthanide-based resonance energy transfer techniques such as HTRF (19) have become the standard for high-performance assays in GPCR activation, mainly due to the millisecond lifetime of the lanthanide excited state and the possibility to reduce cellular background emission (20–22). An interesting phenomenon of luminescent lanthanide complexes is that, due to their elevated millisecond excited-state lifetimes, collisional quenching of their luminescence by acceptor chromophores occurs at low concentrations. This DERET (23–25) phenomenon has already been used to detect electrostatic interactions on proteins (26, 27) and to monitor translocation of lanthanide complexes (10, 28). DERET depends on, among other things, the acceptor concentration and the shortest possible proximity that can occur between the donor and acceptor (23). The DERET was developed to be an efficient internalization and recycling assay (29). It includes the water-soluble terbium cryptate donor (30), which is kinetically very stable, has an exceptionally bright luminescence when excited at 337 nm, and most importantly, does not exhibit a specific binding to cells (16). The O^6^-benzylguanine moiety is able to covalently label the ST (ST) developed by Keppler et al. (31) and has been used to monitor receptor internalization with fluorescence microscopy using classic fluorophores (31–33). The ST is a derivative of O^6^-guanine nucleotide alkyltransferase, which can covalently react with fluorescent-conjugated benzyl guanine substrates (14). To reduce non-specific labeling due to high substrate concentrations, a methyl-benzamide moiety was added to the benzylguanine group to further enhance its reactivity with the ST protein (34). This modification did not alter the quantum-yield of luminescence relative to the parent compound described by Xu et al. (30). Thus, DERET internalization assay relies on the use of a luminescent terbium cryptate derivative (SNAP-Lumi4-Tb) to label irreversibly the cells expressing the ST-GPCRs. Consequently, an efficient DERET should occur if cells expressing a lanthanide complex-labeled receptor on their surface are incubated with micromolar concentrations of an acceptor. Internalization of labeled receptors will prevent the interaction between donor and acceptor and reduce DERET signal intensity (Figure 1A). The receptor internalization assay includes five steps (Figure 1B).

### Validation of the DERET Internalization Assay on Vasopressin V_1a_ Receptors

**Figure 2:**
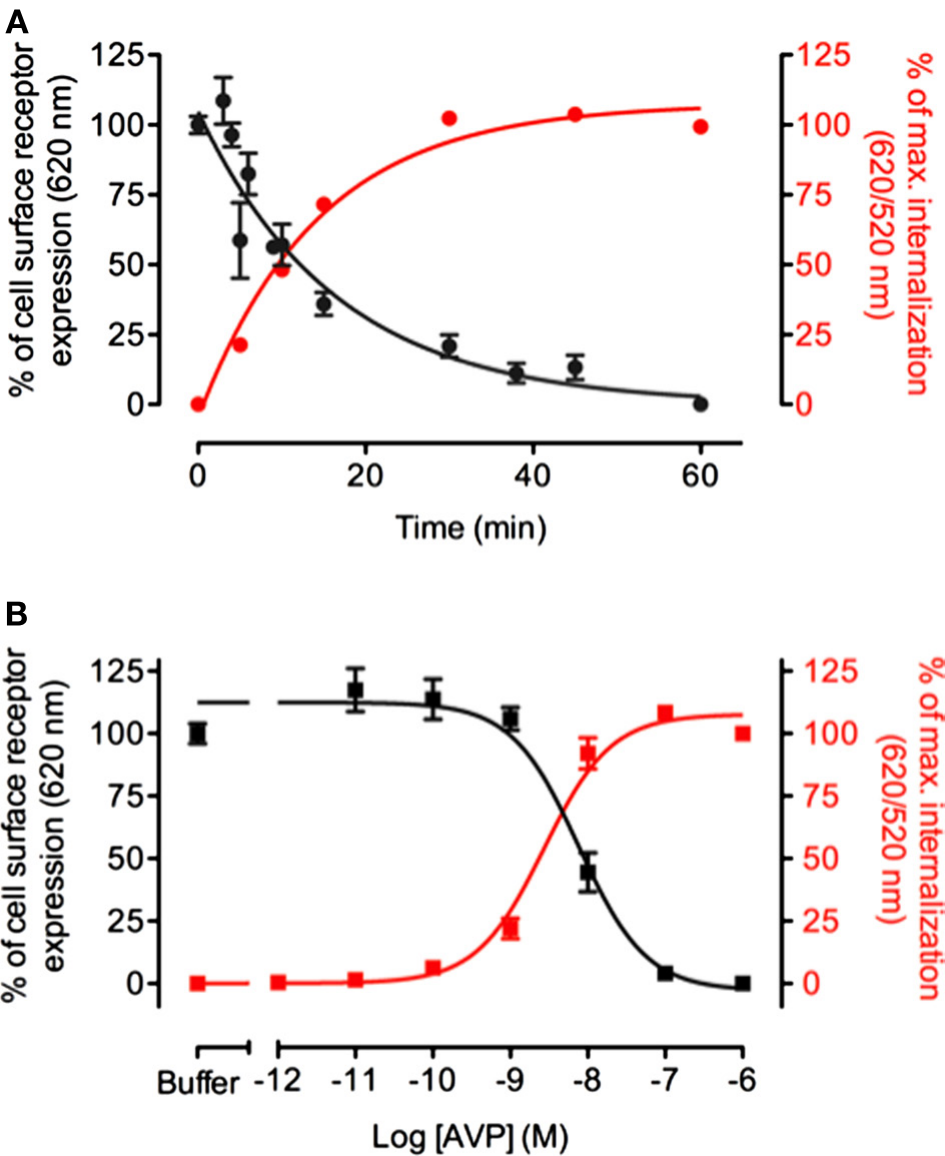
**Comparison between DERET-based internalization assay and measure of receptor cell surface expression**. **(A)** V1a internalization was monitored over time in response to 1 μM of AVP or **(B)** in the presence of increasing concentrations of AVP for 1 h at 37°C. Analysis was performed side-by-side using DERET assay (red line and right *Y* axis) and by measuring receptor expression levels at the cell surface after stimulation by AVP (black line and left *Y* axis) as described in the Section “Materials and Methods.” The data are mean ± SD from three independent experiments performed in triplicate.

To validate the strategy, we compared DERET to a classic method, which consists in evaluating receptors remaining at the cell surface after agonist stimulation. HEK-293 cells stably expressing the ST-vasopressin V_1a_ receptor were incubated either in the presence of a constant AVP concentration for variable time to define kinetics of receptor internalization or in the presence of increasing concentration of AVP to establish the dose–response internalization curves. In a final step, cells were incubated with the non-cell-permeable SNAP-Lumi4-Tb in order to label exclusively receptors which remain at the cell surface. Because DERET assays consist in monitoring the internalized receptor fraction, AVP stimulation resulted in a dramatic increase of fluorescence 620/520 nm ratio (Figure 2A). By contrast, in the classic method, AVP stimulation resulted in a decrease of the fluorescence signal (Figure 2A), indicating receptor depletion from the cell surface. Fluorescence signals were plotted in function of the duration of the incubation with agonist and fitted with a one exponential decay equation routine. The half-time constants (*t*_1/2_) were respectively 9.86 ± 0.004 min and 11.42 ± 0.009 min. Similarly, variations of the fluorescent signals were investigated in function of various AVP concentrations after 1 h incubation (Figure 2B). EC_50_ values determined after fitting experimental data were 2.7 ± 0.06 and 7.7 ± 0.11 nM for DERET and the classic method, respectively. The consistency of the kinetics time constants and EC_50_ values obtained with the two different techniques proves the validity of the DERET strategy to investigate receptor internalization.

### DERET Assay to Study Receptor Recycling

**Figure 3:**
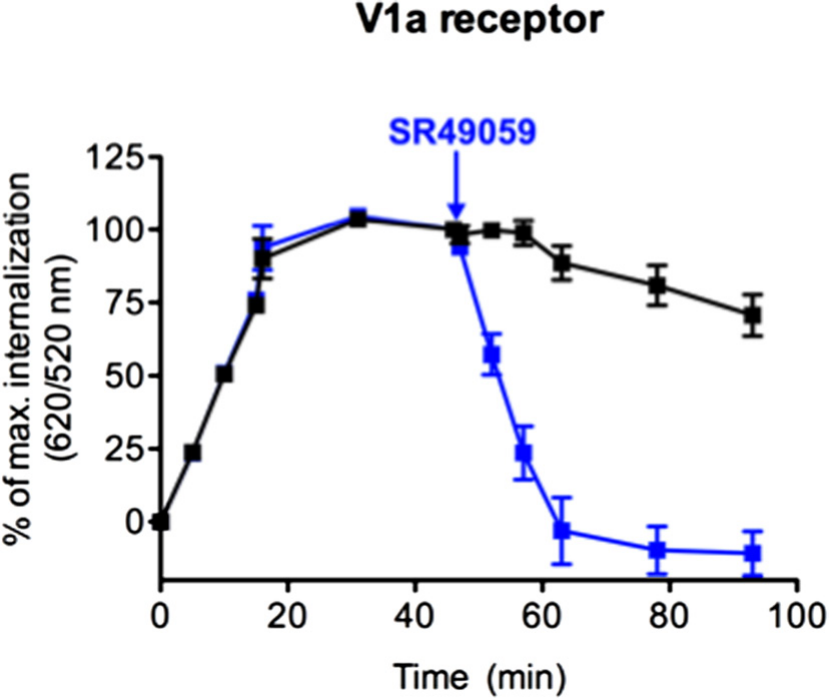
**Real-time internalization and recycling of vasopressin V_1a_ receptor**. Following V_1a_ internalization induced by AVP (1 μM) for 45 min, an excess of the antagonist SR49059 (10 μM) was added (at the time indicated by arrow) to stop internalization and to induce receptor recycling. Black line represents V_1a_ internalization induced by AVP (increase 620/520 ratios). Blue line represents V_1a_ internalization and recycling illustrated by an increase and a decrease in 620/520 ratios, respectively. Percent of ratios were plotted as a function of time and data represent mean ± SD from two independent experiments carried out in triplicates.

Once internalized, receptors can either be degraded or rapidly recycled at the cell surface like the V_1a_ receptor in response to AVP (35). To investigate this recycling, the internalization profile after agonist stimulation was compared to that upon the addition of an antagonist, in a second step. After the addition of an excess of SR49059 (10 μM), a selective antagonist of V_1a_ receptor, the fluorescence ratio decreased to a level comparable to the one in non-stimulated cells (Figure 3). This decrease reflects the re-targeting of labeled ST-V_1a_ receptors at the cell surface thus demonstrating the recycling of the V_1a_ receptor. DERET is therefore a reliable strategy to follow agonist-induced receptor internalization and recycling.

### DERET Assay Allows Characterizing Ligand Properties

**Figure 4:**
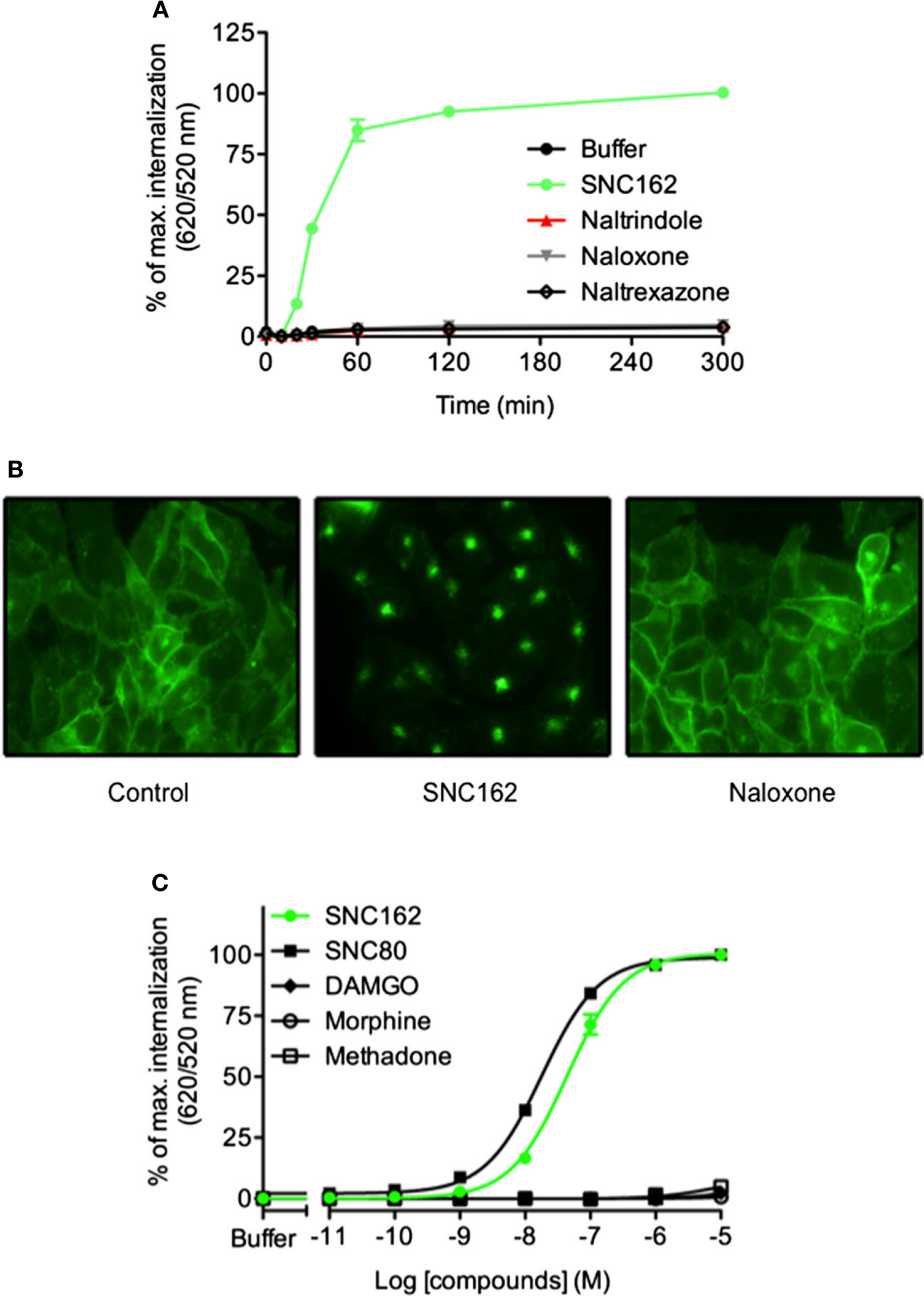
**DERET assay for identification of biased ligand of the **δ** opioïd receptor**. **(A)** ST-δ receptor receptor internalization in the presence of the indicated δ receptor-ligands (10 μM) was measured after first incubating the plate at 4°C (*t* = 0) and then transferring it to room temperature for 5 h. Data represent mean ± SD from one representative experiment carried out in triplicates **(B)** Wide field fluorescence microscopy imaging of ST-δ receptor internalization. ST-δ receptor cells labeled with O^6^-Benzylguanine-Alexa-488 were treated with SNC162 (10 μM) or naloxone (10 μM) for 30 min at room temperature. Receptor internalization was visualized by fluorescence microscopy. Cells expressing labeled ST-δ receptor incubated in the presence of buffer were used as a negative control. **(C)** Dose–response curves analysis of δ receptor. ST-δ receptor expressing cells were incubated with indicated concentrations of SNC162 (closed green circle), SNC80 (closed square), morphine (open circle), methadone (open square), DAMGO (closed triangle) and internalization measured after 1 h at 37°C Data represent mean ± SD from three independent experiments carried out in triplicates and fitted using non-linear regression dose–response log [ligand] versus response with three parameters.

Ligands can differ in their ability to activate distinct signaling cascades through the same receptor. This phenomenon is named functional selectivity or biased signaling. Recent studies have shown that the propensities of GPCR ligands to promote internalization do not necessarily correlate with agonist efficacy. For example, when considering opioïd receptor, some ligands can induce rapid receptor internalization while others such as morphine do not (36). We analyzed δ opioïd receptor internalization using CHO cells stably expressing the ST-receptor (17). After optimizing the labeling of the receptor and signal measurement conditions (Figure S1 in Supplementary Material), we demonstrated that SNC162 induced a dramatic receptor internalization while antagonists (naltrindole, naloxone, and naltrexazone) did not change the fluorescence ratio (Figure 4). Expression of the δ receptor at the cell membrane and its internalization were confirmed by fluorescence microscopy (Figure 4B). We next performed dose–responses experiments with some opioïd receptors agonists to analyze their functional selectivity. We found that SNC162 and SNC80 promoted ST-δ receptor internalization in a dose-dependent manner (Figure 4C). EC_50_ was 43.9 ± 0.04 nM for SNC162 and 18 ± 0.02 nM for SNC80 (Figure 4C), values, which are similar to the ones reported with other methods (37, 38). In contrast, morphine and methadone did not trigger receptor internalization although they are full agonists of δ opioïd receptor for the G_i/o_ signaling pathway thereby demonstrating that they are biased agonists. As expected, the specific μ receptor agonist, DAMGO did not increase the fluorescence ratio, proving the specificity of the assay. Thus, DERET assay can be a relevant strategy to screen for biased agonists on internalization pathway.

### Analyses by DERET of Agonist-Independent GPCR Internalization

**Figure 5:**
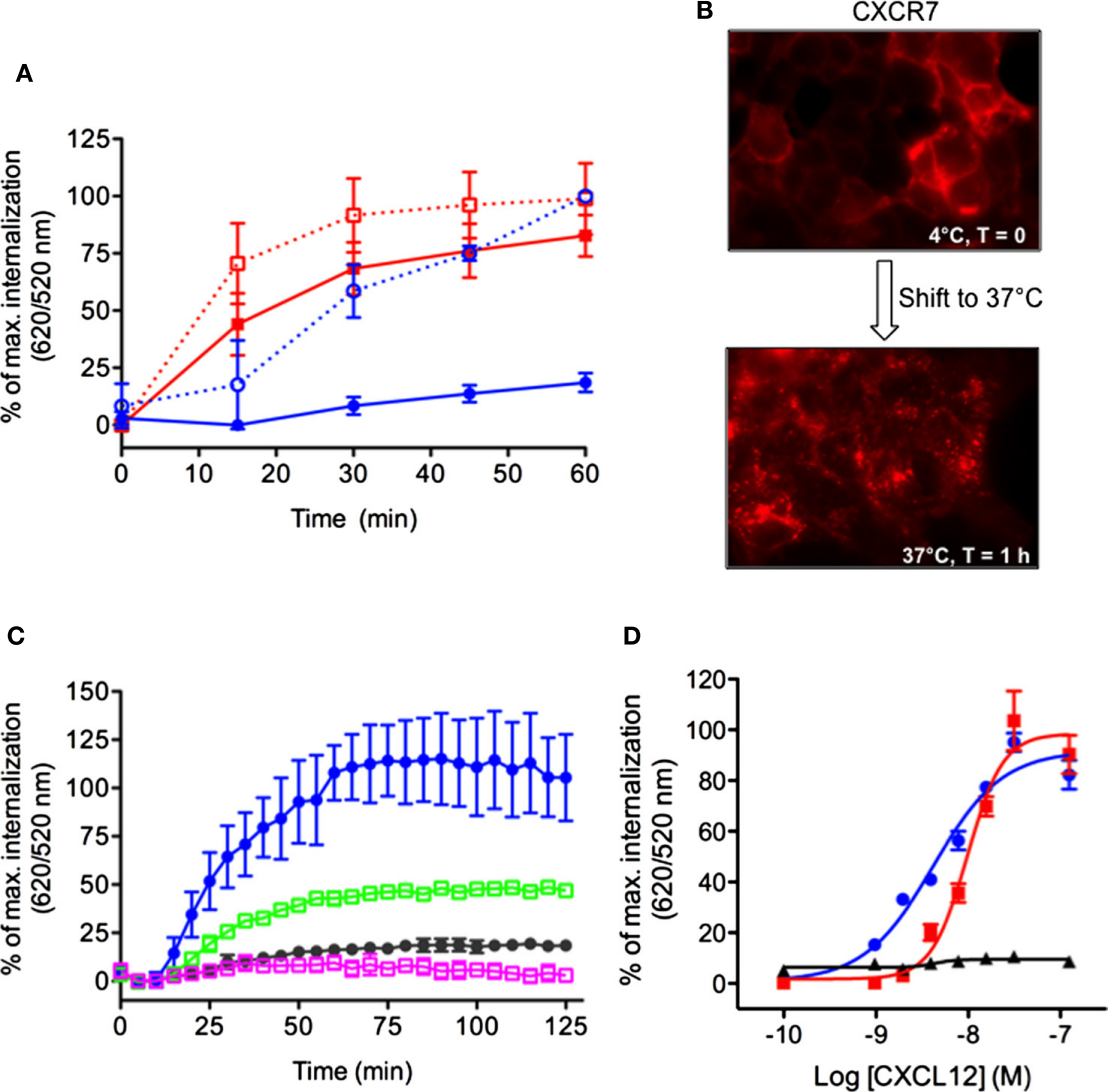
**Simultaneous detection of constitutive and CXCL12-induced internalization of CXCR4 and CXCR7 chemokine receptors**. **(A)** Kinetic analysis of receptor internalization in HEK-293 cells stably expressing ST–CXCR4 or ST–CXCR7 and labeled with SNAP-Lumi4^®^-Tb. At the end of the labeling period, cells were transferred to 37°C (*t* = 0) and internalization was monitored in the presence or absence of 200 nM CXCL12. Results (mean ± SEM) were from three independent experiments performed in duplicate. **(B)** Constitutive internalization of ST–CXCR7 visualized by fluorescence microscopy. Cells expressing ST–CXCR7 were labeled with SNAP-red substrate at 4°C for 1 h and then were either held at 4°C for 1 h (upper panel) or transferred to 37°C for 1 h (lower panel) before being analyzed. **(C)** HEK-293 cells stably expressing ST–CXCR4 were incubated with buffer or CXCL12 (200 nM) alone or with 25 μM AMD3100 (a selective CXCR4 antagonist) or 25 μM chalcone 4 (a CXCL12 antagonist) for 1 h at 4°C. Internalization was then monitored at 37°C for 2 h. Results (mean ± SEM) were from three independent experiments. **(D)** Doses response curves of CXCR4, CXCR7, and CXCR7ΔCter internalization induced by CXCL12. Cells transiently expressing ST–CXCR4 (blue line), ST–CXCR7 (red line), or ST–CXCR7ΔCter (black line) were labeled with SNAP-Lumi4-Tb. Internalization was then measured in the presence of various CXCL12 concentrations for 1 h at 37°C. Data are fitted using non-linear regression dose–response log [agonist] versus response with four parameters. Results (mean ± SEM) were from three independent experiments performed in duplicate.

We sought to extend the DERET internalization assay to other receptors including those which display constitutive internalization. To do so, we compared the internalization profiles of the CXCR7 and CXCR4 receptors, which share the same agonist (CXCL12) but respond to it differently (15, 39–41). CXCL12 is required during embryonic development and regulates many pathophysiological processes in adults (42) but the direct contribution of CXCR7 to CXCL12 functions remains largely unknown (43). In contrast to CXCR4, CXCR7 does not activate the typical G_αi_ pathways of chemokine receptors but signals through β-arrestins (44). Although both CXCR4 and CXCR7 are internalized following stimulation with CXCL12, CXCR7 also displays an atypical propensity to constitutively internalize. Thus, like other atypical chemokine receptors, CXCR7 is thought to act as a decoy receptor that helps to shape CXCL12 gradients and modulate CXCR4 function (2, 43, 45, 46). We investigated the internalization kinetics of CXCR4 and CXCR7 in HEK-293 cells expressing chimeric receptors bearing ST (ST–CXCR4 or ST–CXCR7). Both fusion proteins were expressed normally and showed no alteration of their functional properties (data not shown). Kinetic analyses of receptor internalization were performed at 37°C in the presence or absence of CXCL12 (200 nM). CXCL12 stimulation greatly increased 620/520 ratio in cells expressing ST–CXCR4 compared to the signal in non-stimulated cells (Figure 5A, blue dashed curve versus blue solid curve, respectively). These data indicate that CXCR4 was internalized solely in the presence of CXCL12. By contrast, we detected a high fluorescence ratio in cells expressing ST–CXCR7 whether or not they were stimulated with CXCL12 (Figure 5A, red dashed curve versus red solid curve, respectively). These results indicate that CXCR7 can undergo ligand-dependent as well as ligand-independent internalization. It is noteworthy that the stimulation with CXCL12 induced a moderate change in the magnitude of the fluorescence ratio and slightly enhanced the CXCR7 internalization rate over time. These data extend previous findings showing that CXCL12 treatment has little effect on endogenous CXCR7 internalization in breast cancer cells (45). The constitutive internalization of the CXCR7 receptor was also confirmed in living cells by fluorescence microscopy after the cell surface-expressed ST-receptors were labeled with the cell-impermeable SNAP-Red fluorescent substrate (Figure 5B). Shifting labeled cells from 4 to 37°C for 1 h caused the labeled-receptors to become highly concentrated intracellularly, confirming the propensity of the CXCR7 receptor to internalize in a ligand-independent manner (Figure 5B). CXCL12-induced ST–CXCR4 internalization was inhibited by AMD3100, a specific CXCR4 antagonist (47) and by chalcone 4, a CXCL12 neutraligand (48), which provided further evidence that this internalization was specific to CXCL12-mediated receptor activation (Figure 5C, green and purple squares, respectively). CXCL12 stimulation of cells expressing either ST–CXCR4 or ST–CXCR7 promoted a dose-dependent receptor internalization (Figure 5D), with EC_50_ values of 4.15 ± 0.07 nM for ST–CXCR4 and 9.7 ± 0.05 nM for ST–CXCR7, both of which agree with previous findings (15, 49, 50).

**Figure 6:**
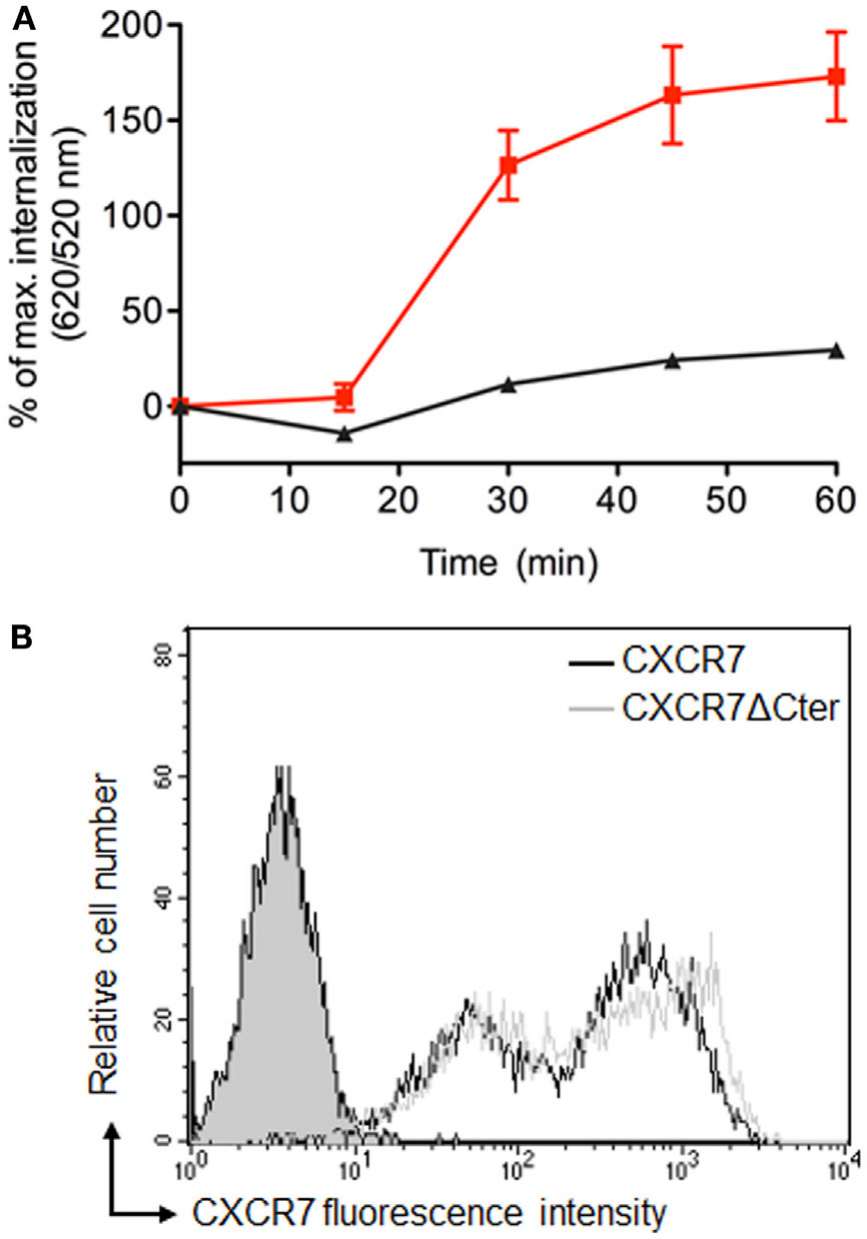
**The C-terminus of CXCR7 is essential for internalization**. **(A)** Kinetic analysis of constitutive internalization of ST–CXCR7 (wild type; red curve) and ST–CXCR7ΔCter (lacking the C-terminus; black curve). HEK-293T cells transiently expressing one type of receptor were labeled with SNAP-Lumi4^®^-Tb. At the end of the labeling period (*t* = 0), internalization was followed over time at 37°C and plotted as a function of time. Results (mean ± SEM) were from three independent experiments performed in duplicate. **(B)** Cell surface expression levels of CXCR7 receptors in HEK-293T cells transiently expressing Flag-ST–CXCR7 or Flag-ST–CXCR7ΔCter. Representative flow cytometry profiles of ST–CXCR7 (black histogram) and ST–CXCR7ΔCter expression (light gray histogram) were determined using anti-CXCR7 antibody. Filled gray histograms show staining with isotype control mAb. Receptor expression is shown as mean fluorescence intensity.

β-arrestins bind to the carboxyl terminus domain (C-ter) of CXCR7, which is essential for CXCL12-induced receptor internalization (39, 44, 51). Analyses performed in cells transiently expressing labeled receptors confirmed that ST–CXCR7 was constitutively internalized over time (Figure 6A, red curve) but that internalization of a receptor lacking the C-ter domain, ST–CXCR7ΔCter, was almost completely abolished (Figures 5D and 6A, black curve). Flow cytometry analysis showed that similar amounts of ST–CXCR7ΔCter and ST–CXCR7 proteins were present at the cell surface of transfected cells, thus excluding a defect in ST–CXCR7ΔCter expression (Figure 6B). The DERET assay allows investigating ligand-independent (constitutive) and -dependent internalization.

### DERET Assay for High-Throughput Screening

**Figure 7:**
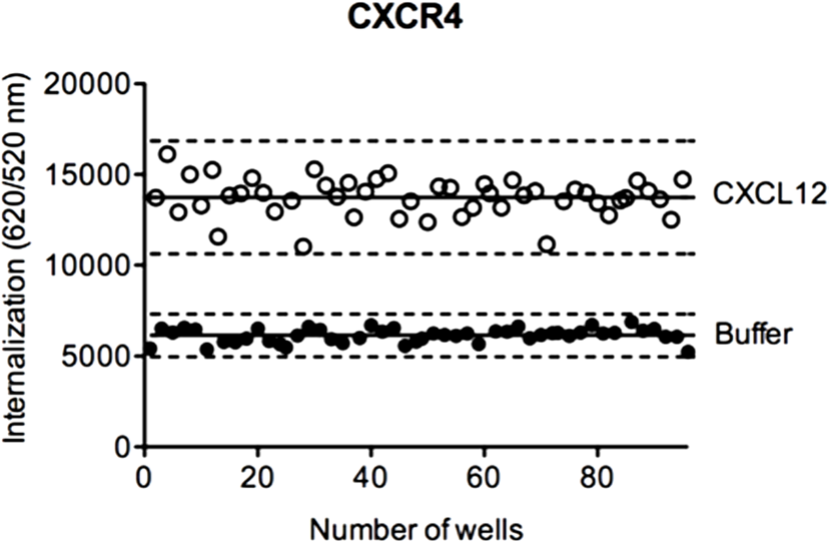
***Z***′**-factor determination in CXCR4 internalization assays**. HEK-293 cells stably expressing ST–CXCR4 were labeled with SNAP-Lumi4^®^-Tb. At the end of the labeling period (*t* = 0), cells were incubated with an excess of acceptor and stimulated or not with 200 nM of CXCL12. Internalization of CXCR4 was followed at 37°C for 1 h as described in the Section “Materials and Methods.” The *Z*′-factor was obtained according to the published methods by Zang *et al*. (52). The scatter plot represents the 48-well positive controls data (with CXCL12, open circle) and the 48-well negative control data (with buffer, closed circle). Solid lines represent the mean internalization signal and dashed lines represent three SD above and below.

To evaluate the suitability of DERET assay for HTS, we determined the *Z*′-factor, a performance indicator that takes into account both signal dynamic range and variation of experimental data (52). In HTS, a *Z*′-factor value between 0 and 0.5 is considered as acceptable. We calculated the *Z*′-factor value for the DERET assay based on CXCR4 internalization in ST–CXCR4 stably expressing cells stimulated with CXCL12 (Figure 7). We found a *Z*′-factor value of 0.45, which demonstrated the soundness of the DERET assay for screening compounds that can potentially modulate receptor internalization and expression at the cell surface.

### Extending DERET Assay to Class C GPCRs

**Figure 8:**
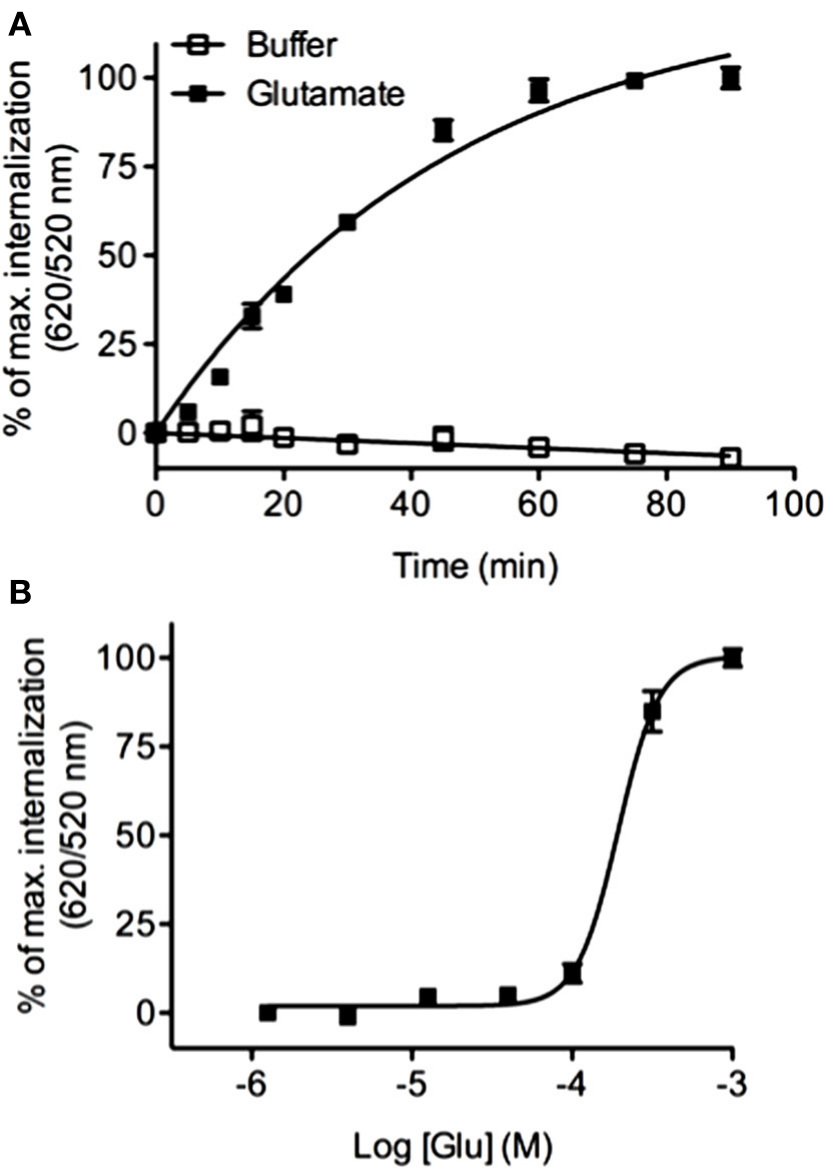
**Real-time internalization of mGluR5**. **(A)** ST-mGluR5 expressed at the cell surface were labeled with SNAP-Lumi4-Tb. Cells were subsequently incubated with an excess of acceptor in the presence of 1 μM of glutamate (closed square) or in absence of agonist (open square). Internalization of mGluR5 was measured at 37°C over time. Data represent mean ± SD from three independent experiments performed in duplicate. Data were fitted using one phase association equation. **(B)** Dose–response curve for mGluR5 in response to glutamate. Cells were stimulated for 1 h with increasing concentrations of glutamate and internalization was monitored. Data represent mean ± SD from three independent experiments performed in triplicate and were fitted using non-linear regression dose–response log (agonist) versus response with three parameters.

Previous studies have reported that the class C mGluR5 GPCR undergoes a rapid internalization in response to glutamate (53). We analyzed mGluR5 internalization by performing kinetic and dose–response experiments using HEK-293 cells stably expressing the ST-receptor. We found that the glutamate agonist was able to promote a time (Figure 8A) and concentration dependent (Figure 8B) internalization of mGluR5 (EC_50_ = 193.3 ± 0.03 μM) thus extending the DERET assay to class C GPCR.

## Conclusion

In conclusion, we provided compiling evidence that the DERET strategy is offering many advantages compared to other internalization assays and constitutes an attractive technology to monitor receptors internalization: (i) The assay is compatible with various receptors models as shown here for receptors of amino-acids such as mGluR5 receptors, of peptides such as vasopressin or opioïd receptors, of proteins such as CXCR4 and CXCR7 receptors and for receptors such as CXCR7 undergoing a ligand-independent internalization and finally previously for receptors of bioamine (54); (ii) the technique is very easy to implement since it is based on a single labeling step by contrast to other conventional techniques based on multiple labeling steps separated by cold acid washing steps; (iii), the method is sensitive due to the use of cryptate of terbium, Lumi4-Tb. Its long lifetime allows to pass over the short-lived fluorescence resulting from auto-fluorescence of the biological samples or medium and directly excite the acceptor, dramatically decreasing the signal to noise ratio. DERET-based assay can also be extended to other tag-mediated labeling utilizing self-labeling proteins such as CLIP-Tag and Halo-Tag, which are reacting covalently with an exogenously supplied substrate that is linked to different fluorescent dyes thereby multiplying the labeling possibilities; (iv), it is a quantitative method to estimate the amplitude of the internalization process and compatible with kinetics and dose–response experiments, allowing characterization of pharmacological parameters and the identification of biased agonists; (v), the receptor is directly and covalently labeled allowing tracking the receptor itself and not the complex between the receptor and the ligand. This is particularly important to study receptor recycling or degradation since the fusion of endosomes to lysosome induces an acidification of the vesicular medium and the dissociation of the ligand/receptor complex; (vi), the reduced number of steps of the DERET strategy and the high signal/noise ratio make this assay suitable for HTS as supported by the *Z*′-value characterized in this study. The major drawback of DERET is that it relies on chimeric receptors and is not compatible with the investigation of endogenous receptors. Nevertheless, to our knowledge, no effect of self-labeling domains (ST, CLIP-tag, HaloTag) have been reported so far on the pharmacology of the receptors (14, 16, 55–57). It is also noteworthy that DERET does not allow an absolute but a relative quantification, making difficult the comparison of the DERET signal obtained on different receptor models. Moreover, in the time course of an experiment, neo-synthesized receptors that have reached the cell surface will not be considered since they have not been labeled. This limitation could eventually impact the accuracy of the quantification of the internalization in case of large over-expression of the receptor.

To conclude, we have shown that DERET is a relevant strategy to study internalization kinetics, dose–response internalization, receptor recycling, receptor constitutive internalization and to screen for biased ligand. DERET is therefore of broad interest to study GPCR internalization.

## Author Contributions

AL initiated and performed most of the experiments and wrote the manuscript. MC, AJ-R, LK, SB, JZ, DM, participated in the experiments and analyses, ET, KB, and LP participated in project support. FB and TD initiated and supported the project and participated to the analyses and to the writing of the manuscript. All authors critically reviewed the manuscript and approved the final version.

## Disclaimer

Lumi4 and Tag-lite are registered trademarks of Lumiphore and Cisbio Bioassays, respectively.

## Conflict of Interest Statement

The authors declare that the research was conducted in the absence of any commercial or financial relationships that could be construed as a potential conflict of interest.

## References

[1] Bockaert J Pin J-P. Molecular tinkering of G protein-coupled receptors: an evolutionary success. EMBO J (1999) 18(7):1723–9. 10.1093/emboj/18.7.1723 10202136 PMC1171258

[2] Ferguson SSG Evolving concepts in G protein-coupled receptor endocytosis: the role in receptor desensitization and signaling. Pharmacol Rev (2001) 53:1–24. 11171937

[3] Calebiro D Nikolaev VO Persani L Lohse MJ. Signaling by internalized G-protein-coupled receptors. Trends Pharmacol Sci (2010) 31:221–8. 10.1016/j.tips.2010.02.002 20303186

[4] Graham GJ Locati M Mantovani A Rot A Thelen M. The biochemistry and biology of the atypical chemokine receptors. Immunol Lett (2012) 145(1–2):30–8. 10.1016/j.imlet.2012.04.004 22698181

[5] Garippa RJ Hoffman AF Gradl G Kirsch A High-Throughput Confocal Microscopy for β-arrestin Green Fluorescent Protein Translocation G Protein-Coupled Receptor Assays Using the Evotec Opera. In: Inglese J, editor. Measuring Biological Responses with Automated Microscopy (Vol. 414), San Diego, CA: Academic Press (2006). p. 99–120. 10.1016/S0076-6879(06)14007-0 17110189

[6] Eriksen J Bjørn-Yoshimoto WE Jørgensen TN Newman AH Gether U Postendocytic sorting of constitutively internalized dopamine transporter in cell lines and dopaminergic neurons 10.1074/jbc.M110.131003. J Biol Chem (2010) 285(35):27289–301. 10.1074/jbc.M110.131003 20551317 PMC2930728

[7] Hamdan FF Audet M Garneau P Pelletier J Bouvier M High-throughput screening of G protein-coupled receptor antagonists using a bioluminescence resonance energy transfer 1-Based β-Arrestin2 recruitment assay 10.1177/1087057105275344. J Biomol Screen (2005) 10(5):463–75. 10.1177/1087057105275344 16093556

[8] Lam VM Beerepoot P Angers S Salahpour A A novel assay for measurement of membrane-protein surface expression using a beta-lactamase. Traffic (2013) 14(7):778–84. 10.1111/tra.12073 23574269

[9] Hammer MM Wehrman TS Blau HM. A novel enzyme complementation-based assay for monitoring G-protein-coupled receptor internalization. FASEB J (2007) 21(14):3827–34. 10.1096/fj.07-8777com 17942829

[10] Alvarez-Curto E Prihandoko R Tautermann CS Zwier JM Pediani JD Lohse MJ Developing chemical genetic approaches to explore G protein-coupled receptor function: validation of the use of a receptor activated solely by synthetic ligand (RASSL). Mol Pharmacol (2011) 80(6):1033–46. 10.1124/mol.111.074674 21880827 PMC3228535

[11] Ashby MC Ibaraki K Henley JM. It’s green outside: tracking cell surface proteins with pH-sensitive GFP. Trends Neurosci (2004) 27(5):257–61. 10.1016/j.tins.2004.03.010 15111007

[12] Robers MB Binkowski BF Cong M Zimprich C Corona C McDougall M A luminescent assay for real-time measurements of receptor endocytosis in living cells. Anal Biochem (2015) 489:1–8. 10.1016/j.ab.2015.08.005 26278171

[13] Fisher GW Adler SA Fuhrman MH Waggoner AS Bruchez MP Jarvik JW. Detection and quantification of beta2AR internalization in living cells using FAP-based biosensor technology. J Biomol Screen (2010) 15(6):703–9. 10.1177/1087057110370892 20488980

[14] Maurel D Comps-Agrar L Brock C Rives ML Bourrier E Ayoub MA Cell-surface protein-protein interaction analyses with time-resolved FRET and snap-tag technologies: application to GPCR oligomerization. Nat Methods (2008) 5:561–7. 10.1038/nmeth.1213 18488035 PMC2642604

[15] Levoye A Balabanian K Baleux F Bachelerie F Lagane B CXCR7 heterodimerizes with CXCR4 and regulates CXCL12-mediated G protein signaling 10.1182/blood-2008-12-196618. Blood (2009) 113(24):6085–93. 10.1182/blood-2008-12-196618 19380869

[16] Doumazane E Scholler P Zwier JM Trinquet E Rondard P Pin JP A new approach to analyze cell surface protein complexes reveals specific heterodimeric metabotropic glutamate receptors. FASEB J (2011) 25(1):66–77. 10.1096/fj.10-163147 20826542

[17] Zwier JM Roux T Cottet M Durroux T Douzon S Bdioui S A fluorescent ligand-binding alternative using Tag-lite(R) technology. J Biomol Screen (2010) 15(10):1248–59. 10.1177/1087057110384611 20974902

[18] Faklaris O Cottet M Falco A Villier B Laget M Zwier JM Multicolor time-resolved Forster resonance energy transfer microscopy reveals the impact of GPCR oligomerization on internalization processes. FASEB J (2015) 29(6):2235–46. 10.1096/fj.14-260059 25690655

[19] Mathis G. Probing molecular interactions with homogeneous techniques based on rare earth cryptates and fluorescence energy transfer. Clin Chem (1995) 41:1391–7. 7656455

[20] Olofsson L Felekyan S Doumazane E Scholler P Fabre L Zwier JM Fine tuning of sub-millisecond conformational dynamics controls metabotropic glutamate receptors agonist efficacy. Nat Commun (2014) 5:5206. 10.1038/ncomms6206 25323157

[21] Degorce F Card A Soh S Trinquet E Knapik GP Xie B HTRF: a technology tailored for drug discovery – a review of theoretical aspects and recent applications. Curr Chem Genomics (2009) 3:22–32. 10.2174/1875397300903010022 20161833 PMC2802762

[22] Trinquet E Bouhelal R Dietz M Monitoring Gq-coupled receptor response through inositol phosphate quantification with the IP-One assay. Expert Opin Drug Discovery (2011) 6(10):981–94. 10.1517/17460441.2011.608658 22646860

[23] Thomas DD Carlsen WF Stryer L. Fluorescence energy transfer in the rapid-diffusion limit. Proc Natl Acad Sci USA (1978) 75:5746–50. 10.1073/pnas.75.12.5746 16592590 PMC393050

[24] Meares CF Yeh SM Stryer L Exchange interaction contribution to energy transfer between ions in the rapid diffusion limit. J Am Chem Soc (1981) 103:1607–9. 10.1021/ja00396a073

[25] Koresawa M Kikuchi K Mizukami S Kojima H Urano Y Higuchi T Development of a time-resolved fluorometric detection system using diffusion-enhanced energy transfer. Anal Chem (2000) 72:4904–7. 10.1021/ac000356t 11055707

[26] Meltzer RH Lurtz MM Wensel TG Pedersen SE Nicotinic acetylcholine receptor channel electrostatics determined by diffusion enhanced luminescence energy transfer. Biophys J (2006) 91:1315–24. 10.1529/biophysj.106.081448 16751249 PMC1518635

[27] Northrup SH Wensel TG Meares CF Wendoloski JJ Matthew JB. Electrostatic field around cytochrome c: theory and energy transfer experiment. Proc Natl Acad Sci USA (1990) 87:9503–7. 10.1073/pnas.87.23.9503 2174564 PMC55195

[28] Zheng Q Dai H Merritt ME Malloy C Pan CY Li WH. A new class of macrocyclic lanthanide complexes for cell labeling and magnetic resonance imaging applications. J Am Chem Soc (2005) 127:16178–88. 10.1021/ja054593v 16287307

[29] Roed SN Wismann P Underwood CR Kulahin N Iversen H Cappelen KA Real-time trafficking and signaling of the glucagon-like peptide-1 receptor. Mol Cell Endocrinol (2014) 382(2):938–49. 10.1016/j.mce.2013.11.010 24275181

[30] Xu J Corneillie TM Moore EG Law GL Butlin NG Raymond KN Octodentate cages of Tb(III) 2-hydroxyisophtalamides: a new standard for luminescence lanthanide labels. J Am Chem Soc (2011) 133:19900–10. 10.1021/ja2079898 22010878 PMC3240577

[31] Keppler A Gendreizig S Gronemeyer T Pick H Vogel H Johnsson K. A general method for the covalent labeling of fusion proteins with small molecules in vivo. Nat Biotechnol (2003) 21:86–9. 10.1038/nbt765 12469133

[32] Komatsu T Johnsson K Okuno H Bito H Inoue T Nagano T Real-time measurements of protein dynamics using fluorescence activation-coupled protein labeling method. J Am Chem Soc (2011) 133(17):6745–51. 10.1021/ja200225m 21473619

[33] Cole NB Donaldson JG Releasable SNAP-tag probes for studying endocytosis and recycling. ACS Chem. Biol (2012) 7:464–9. 10.1021/cb2004252 PMC3307366 22216966

[34] Zwier JM Bazin H Lamarque L Mathis G. Luminescent lanthanide cryptates: from the bench to the bedside. Inorg Chem (2014) 53(4):1854–66. 10.1021/ic402234k 24392868

[35] Innamorati G Le Gouill C Balamotis M Birnbaumer M. The long and the short cycle. Alternative intracellular routes for trafficking of G-protein-coupled receptors. J Biol Chem (2001) 276(16):13096–103. 10.1074/jbc.M009780200 11150299

[36] Yang J Yang H Du X Ma Q Song J Chen M Morphine and DAMGO produce an opposite effect on presynaptic glutamate release via different downstream pathways of mu opioid receptors in the basolateral amygdala. Neuropharmacology (2014) 86:353–61. 10.1016/j.neuropharm.2014.08.021 25196735

[37] Charfi I Nagi K Mnie-Filali O Thibault D Balboni G Schiller PW Ligand- and cell-dependent determinants of internalization and cAMP modulation by delta opioid receptor (DOR) agonists. Cell Mol Life Sci (2014) 71(8):1529–46. 10.1007/s00018-013-1461-7 24022593 PMC3952036

[38] Pradhan AAA Becker JA Scherrer G Tryoen-Toth P Filliol D Matifas A Delta opioid receptor internalization controls behavioral effects of agonists. PLoS One (2009) 4(5):e5425 10.1371/journal.pone.0005425 19412545 PMC2672171

[39] Balabanian K Lagane B Infantino S Chow KY Harriague J Moepps B The chemokine SDF-1/CXCL12 binds to and signals through the orphan receptor RDC1 in T lymphocytes 10.1074/jbc.M508234200. J Biol Chem (2005) 280(42):35760–6. 10.1074/jbc.M508234200 16107333

[40] Boldajipour B Mahabaleshwar H Kardash E Reichman-Fried M Blaser H Minina S Control of chemokine-guided cell migration by ligand sequestration. Cell (2008) 132(3):463–73. 10.1016/j.cell.2007.12.034 18267076

[41] Naumann U Cameroni E Pruenster M Mahabaleshwar H Raz E Zerwes HG CXCR7 functions as a scavenger for CXCL12 and CXCL11. PLoS One (2010) 5(2):e9175. 10.1371/journal.pone.0009175 20161793 PMC2820091

[42] Kryczek I Wei S Keller E Liu R Zou W Stroma-derived factor (SDF-1/CXCL12) and human tumor pathogenesis 10.1152/ajpcell.00406.2006. Am J Physiol Cell Physiol (2007) 292(3):C987–95. 10.1152/ajpcell.00406.2006 16943240

[43] Freitas C Desnoyer A Meuris F Bachelerie F Balabanian K Machelon V. The relevance of the chemokine receptor ACKR3/CXCR7 on CXCL12-mediated effects in cancers with a focus on virus-related cancers. Cytokine Growth Factor Rev (2014) 25(3):307–16. 10.1016/j.cytogfr.2014.04.006 24853339

[44] Rajagopal S Kim J Ahn S Craig S Lam CM Gerard NP Beta-arrestin- but not G protein-mediated signaling by the “decoy” receptor CXCR7. Proc Natl Acad Sci U S A (2009) 107:628–32. 10.1073/pnas.0912852107 20018651 PMC2818968

[45] Luker KE Steele JM Mihalko LA Ray P Luker GD. Constitutive and chemokine-dependent internalization and recycling of CXCR7 in breast cancer cells to degrade chemokine ligands. Oncogene (2010) 29(32):4599–610. 10.1038/onc.2010.212 20531309 PMC3164491

[46] Thelen M Thelen S. CXCR7, CXCR4 and CXCL12: an eccentric trio? J Neuroimmunol (2008) 198(1–2):9–13. 10.1016/j.jneuroim.2008.04.020 18533280

[47] De Clercq E. The AMD3100 story: the path to the discovery of a stem cell mobilizer (Mozobil). Biochem Pharmacol (2009) 77(11):1655–64. 10.1016/j.bcp.2008.12.014 19161986

[48] Hachet-Haas M Balabanian K Rohmer F Pons F Franchet C Lecat S Small neutralizing molecules to inhibit actions of the chemokine CXCL12. J Biol Chem (2008) 283(34):23189–99. 10.1074/jbc.M803947200 18556651

[49] Di Salvo J Koch GE Johnson KE Blake AD Daugherty BL DeMartino JA The CXCR4 agonist ligand stromal derived factor-1 maintains high affinity for receptors in both Galpha(i)-coupled and uncoupled states. Eur J Pharmacol (2000) 409(2):143–54. 10.1016/S0014-2999(00)00846-3 11104827

[50] Gravel SP Malouf C Boulais PE Berchiche YA Oishi S Fujii N The peptidomimetic CXCR4 antagonist TC14012 recruits β-Arrestin to CXCR7 10.1074/jbc.C110.147470. J Biol Chem (2010) 285(49):37939–43. 10.1074/jbc.C110.147470 20956518 PMC2992227

[51] Luker KE Gupta M Steele JM Foerster BR Luker GD. Imaging ligand-dependent activation of CXCR7. Neoplasia (2009) 11:1022–35. 10.1593/neo.09724 19794961 PMC2745668

[52] Zhang JH Chung TD Oldenburg KR. A simple statistical parameter for use in evaluation and validation of high throughput screening assays. J Biomol Screen (1999) 4(2):67–73. 10.1177/108705719900400206 10838414

[53] Fourgeaud L Bessis AS Rossignol F Pin JP Olivo-Marin JC Hémar A. The metabotropic glutamate receptor mGluR5 is endocytosed by a clathrin-independent pathway. J Biol Chem (2003) 278(14):12222–30. 10.1074/jbc.M205663200 12529370

[54] Pou C Mannoury la Cour C Stoddart LA Millan MJ Milligan G. Functional homomers and heteromers of dopamine D2L and D3 receptors co-exist at the cell surface. J Biol Chem (2012) 287(12):8864–78. 10.1074/jbc.M111.326678 22291025 PMC3308812

[55] Loison S Cottet M Orcel H Adihou H Rahmeh R Lamarque L Selective fluorescent nonpeptidic antagonists for vasopressin V(2) GPCR: application to ligand screening and oligomerization assays. J Med Chem (2012) 55(20):8588–602. 10.1021/jm3006146 22984902

[56] Maurel D Kniazeff J Mathis G Trinquet E Pin JP Ansanay H. Cell surface detection of membrane protein interaction with homogeneous time-resolved fluorescence resonance energy transfer technology. Anal Biochem (2004) 329(2):253–62. 10.1016/j.ab.2004.02.013 15158484

[57] Hounsou C Margathe JF Oueslati N Belhocine A Dupuis E Thomas C Time-resolved FRET binding assay to investigate hetero-oligomer binding properties: proof of concept with dopamine D1/D3 heterodimer. ACS Chem Biol (2015) 10(2):466–74. 10.1021/cb5007568 25350273

