# Supplementary material for: A Broad G Protein-Coupled Receptor Internalization Assay that Combines SNAP-Tag Labeling, Diffusion-Enhanced Resonance Energy Transfer, and a Highly Emissive Terbium Cryptate
Source: Front Endocrinol (Lausanne). 2015 Nov 9;6:167. doi: 10.3389/fendo.2015.00167 (PMC4638144; doi:10.3389/fendo.2015.00167)
Supplement: Supplementary file 1 [file presentation_1.pdf]

Figure supp 1

Levoye et al.

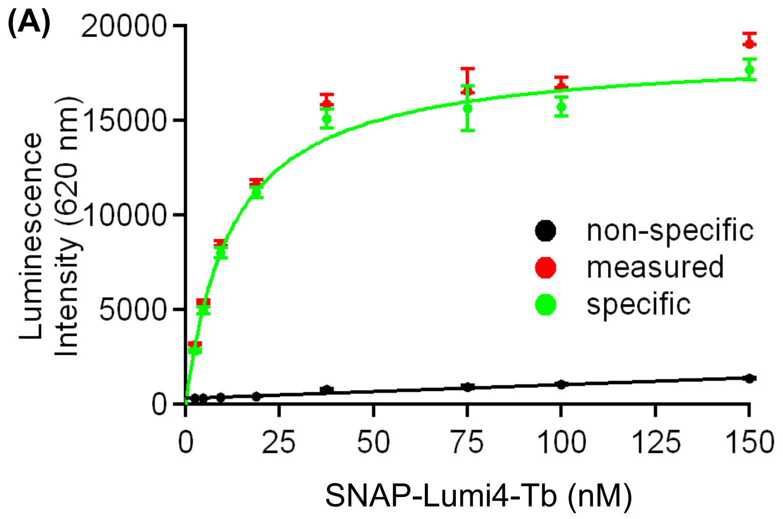

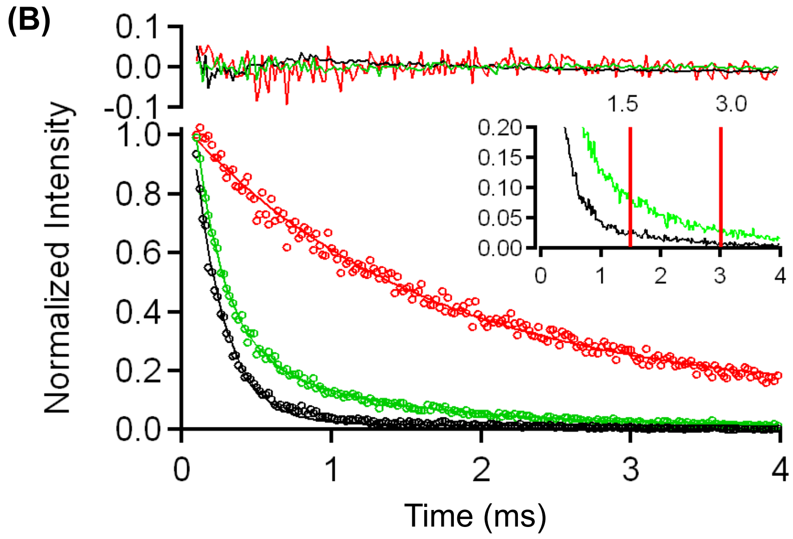

Supplementary Material

Figure S1 : SNAP-Lumi4-Tb labeling and TR-FRET reading parameters

(A) Determination of labeling efficiency with SNAP-Lumi4-Tb in CHO cells stably expressing ST-δ receptor. Non-specific labeling was determined by measuring SNAP-Lumi4-Tb labeling in CHO cells not expressing ST-δ receptor. Specific labeling to ST-δ receptor was calculated by subtracting the non-specific labeling from the measured total labeling in CHO cells stably expressing ST-δ receptor. **(B)** SNAP-Lumi4-Tb donor decay traces at 620 nm in the absence of fluorescein (red points and traces) and after adding fluorescein either with (green dots and traces) or without (black dots and traces) agonist (10 µM SNC-162). Decays were monitored and intensities normalized after 0.1 ms of initial delay. The optimal dynamic range of donor emission in the assay occurred between 1.5 and 3 ms after excitation (see insert).
